# Rereading Habermas in Times of CRISPR-cas: A Critique of and an Alternative to the Instrumentalist Interpretation of the Human Nature Argument

**DOI:** 10.1007/s11673-022-10206-7

**Published:** 2022-09-23

**Authors:** Annett Wienmeister

**Affiliations:** 1 Freie Universität Berlin, Institut für Philosophie, Habelschwerdter Allee 30, 14195 Berlin, Germany; 2 Charité - Universitätsmedizin Berlin, Institut für Geschichte der Medizin und Ethik in der Medizin, Thielallee 71, 14195 Berlin, Germany

**Keywords:** Argument from human nature, Habermas, Germline editing, Genetic engineering

## Abstract

Habermas’s argument from human nature, which speaks in favour of holding back the use of human germline editing for purposes of enhancement, has lately received criticism anew. Prominent are objections to its supposedly genetic essentialist and determinist framework, which underestimates social impacts on human development. I argue that this criticism originates from an instrumentalist reading of Habermas’s argument, which wrongly focuses on empirical conditions and means-ends-relations. Drawing on Habermas’s distinction of a threefold use of practical reason, I show how an alternative—the ethical—reading avoids essentialist and determinist objections by addressing an existential level of sense making. I present three reasons that speak in favour of the ethical reading and I demonstrate how it incorporates social aspects of character formation. Habermas’s account therefore offers exactly what the critics claim is missing. The paper concludes with a conceptual challenge that the ethical reading has to face within Habermas’s overall approach to genetic engineering.

## Introduction

Recent developments in human germline editing (i.e., with the CRISPR-cas system)^1^ move the ethical debate from a rather abstract level about the general tenability of the technology to a more nuanced discussion about ethical implications of specific forms of application, such as interventions for monogenetic and polygenetic diseases or for purposes of enhancement.^2^ This might be one of the reasons why the argument from human nature brought forth by Jürgen Habermas in his *The Future of Human Nature* (2003) (*FHN*) has gained renewed attention. Habermas argues that the natural contingency of the biological genetic setup of humans plays a fundamental role for their self-understanding as moral agents. This is why one should proceed with caution when intentionally introducing changes to the human genome, especially for the purpose of enhancement.

Lately, this argument has received criticism anew. One major objection questions the supposed relationship between genetic contingency of natural procreation and our self-understanding as autonomous and equal members of a moral community and it imputes genetic essentialism and determinism to Habermas’s account. It furthermore accuses it of neglecting social aspects of character formation (Herissone-Kelly 2012; Murphy 2014; Morar 2015; Rothenfluch 2017; Feeney 2019). I will analyze this criticism and show that it originates from a specific kind of reading of the argument, which I will call the instrumentalist reading. This reading builds on evaluating empirical conditions, in this case concerning the impact genes have on personality formation, and it discusses germline editing as an (in)adequate means for achieving ends that are not themselves debated (e.g., enhancement). I will argue that this reading is not convincing and that there is a more adequate interpretation. Drawing on a threefold distinction of practical reason and reasoning, which Habermas introduces in his article “On the pragmatic, the ethical, and the moral employments of practical reason” (2001), I will speak in favour of another reading of the argument, which I call the ethical reading. An ethical reading is not concerned with finding or avoiding specific means to undebated ends but more fundamentally with finding out which ends to pursue in the first place; therefore, it alludes to questions of personal identity and self-understanding at another level of sense making, namely an existential level. The specific degree to which the moral self-understanding of humans is determined by certain genes is not relevant for that matter. Consequently, the ethical reading avoids objections of genetic essentialism and determinism. What is more, Habermas understands the process of identity formation of individuals to rely heavily on processes of socialization within a linguistic community. The ethical reading incorporates this kind of social impact on character formation and thereby offers exactly what is supposed to be missing, according to the critics.

In what follows, I will take a closer look at the two different readings of Habermas’s argument from human nature. The paper begins with a short introduction of his notion of human nature and his general approach to human genetic engineering in *FHN*. In a second step, I will present the threefold use of practical reason and I will show how this distinction by Habermas helps differentiate between the instrumentalist and the ethical interpretation of his argument. I will then present genetic essentialist and determinist objections to the argument as originating from the instrumentalist reading—a reading that is shown to be inadequate. In a fourth step, I will offer three reasons that speak in favour of the alternative, ethical reading and I map out how social structures and processes figure in this context. The paper concludes by discussing a conceptual tension that arises within the ethical reading.

## Habermas’s Notion of Human Nature and Summary of his Argument From Human Nature

In his *FHN*, Jürgen Habermas addresses the question whether biogenetic engineering in humans is ethically tenable or not. His analysis is concerned mainly with pre-implantation genetic diagnosis and research consuming embryos. It is also—and maybe even more so—applicable to interventions with germline editing because not only do they aim at selecting but also at changing the human genome. Habermas brings forward an argument from human nature according to which one should proceed with caution when introducing changes to the human genome because human nature is supposed to play a fundamental role for the self-understanding of persons as moral agents. The notion of human nature in this argument implies three interconnected meanings: First, it refers to those aspects of human life that are existent without a person actively choosing or promoting it (22), second, it relates to the aspects of “chance” and “contingency” characteristic of biological procreation (28, 72), and third, it relates to the circumstance that the genetic setup of humans is out of their reach (22). Habermas contrasts the concept of human nature as that which is given, contingent, and beyond one’s influence to the concept of “the made”. The latter refers to the fact that humans entertain in actively choosing, planning and conducting various activities (44). The meaning of human nature therefore remains at a rather abstract level and does not characterize certain genetic features or character traits of humans as natural. What is worthy of preservation is thus not human nature in the sense of specific human characteristics but the way these characteristics come about. This will be important when discussing the objection that Habermas’s argument relies on genetic essentialist and determinist assumptions.^3^

Habermas’s argument from nature confers a positive value to human nature in the following sense: he understands the uncontrollability of the genetic makeup in natural reproductive processes to be a condition of the self-understanding of individuals as being free and morally equal persons in society. According to him, it is the “nature-like growth which alone allow[s] us to conceive of ourselves as the authors of our own lives and as equal members of the moral community” (*FHN*, 42). Habermas sees the sole authorship of an individual’s life to be threatened by genetic interventions parents chose for their offspring. By deciding on certain genetically bound characteristics, parents introduce an aspect of intentional design into the parent-child relationship that has formerly not been there. For parents, this equals to a shift from chance to choice; for treated children, it might mean loss of contingency as well as a burdensome awareness of parental expectations (*FHN*, 51). Since germline editing introduces permanent changes to the genome of an individual, gene edited children are thought to have no possibility to distance themselves from the expectations of their parents. Habermas sees a difference here between influences parents have on their children through upbringing and education and the biotechnological shaping of the human body. While the former, though also enormously powerful, might be critically reflected in communicative acts and to some extent rejected through a change of cultural practices, determinations of the germline are irreversible for that specific individual. What is missing here is a way to distance oneself from genetic determination in the space of communicative reason, which is why grown-up children and parents might not be experiencing themselves as equals (*FHN*, 14, 61f.).

A common objection against Habermas’s distinction between education and genetic engineering holds that the threat of a burdensome influence is not exclusively a problem of genetic engineering but applies to education and upbringing as well. I will not focus on this objection here because it changes nothing in the negative evaluation of genetic interventions by Habermas. The possibility of dissonance between children and parents is a challenge in each case per se. For a critical discussion and charitable reading of Habermas’s distinction of parental influence on children through education in contrast to genetic engineering, see Enno Fischer (2016).

Habermas introduces a second distinction, which cuts across the juxtaposition of education and genetic engineering. It is the distinction between therapy and enhancement. According to him, not every kind of genetic engineering is problematic in the above sense, as he does not reject interventions for purposes of therapy. In case of therapy, Habermas grants that a broad consensus with future individuals can be assumed. This is supposed to be different in cases of enhancement, where parents choose the genetic setup of their children according to their individual preferences. I will say more about the distinction between therapy and enhancement below.

## The Threefold Use of Practical Reason as a Source of Different Interpretations of the Argument From Human Nature

In his essay “On the pragmatic, the ethical, and the moral employments of practical reason” (2001), Habermas distinguishes three kinds of meaning the question “What should I do?” might take. Accordingly, he identifies three ways of how to conceive of the problem and three ways of how to justify choices among alternative solutions. First, according to the pragmatic employment of reason, one asks what means are adequate to realize subjective preferences, ends, and values that are themselves not fundamentally scrutinized. Answering the question “What should I do?” is in this case “a matter of settling empirical questions and questions of rational choice,” a matter of finding “suitable technology or a realizable program of action” (2001, 8). Second, according to the ethical employment of reason, one asks more fundamental questions, not only about adequate means to given interests and values but about what values and interests to hold in the first place. Ethical questions concern important value decisions that affect the course of life and therefore involve a “hermeneutical clarification of an individual’s self-understanding,” a critical assessment of one’s own life history and integration in various traditions. The ethical employment of practical reason therefore “operates within the horizon of a life history, in whose traces the individual can discern who he is and who he would like to become” (2001, 8f.). As the ethical employment of reason is concerned with questions of the good life and answers to these questions can differ from individual to individual, conflicts may arise. This is where the third employment of practical reason, its moral use, comes into play. It is not concerned with finding adequate means or choosing values constitutive of one’s identity, it rather asks for a just resolution of a conflict between differing interests of a group of people. It aims at “justification and application of norms that stipulate reciprocal rights and duties” with the overall aim of justice (2001, 9).

Please note that Habermas’s threefold distinction of a pragmatic, an ethical, and a moral use of practical reason has a partial analogy in Immanuel Kant’s distinction of hypothetical and categorical imperatives as introduced in his *Groundwork of the Metaphysics of Morals*. The hypothetical-technical imperative describes rules of skill in the sense that one chooses adequate means in order to achieve individual ends. The hypothetical-pragmatic imperative describes counsels of prudence in the sense that one chooses adequate means in order to achieve a universal end all humans share. Both hypothetical imperatives amount to Habermas’s pragmatic use of reason. Finally, there is the categorical imperative, which commands an action unconditionally without a person presupposing individual or universal ends. This relates to Habermas’s moral use of reason, although he rejects Kant’s theory of an intelligible realm of human agency (2001, 10; *FHN*, 34).

While Habermas refers to Kant’s distinction of the three kinds of imperatives of reason, his terminology differs.^4^ His pragmatic employment of reason matches the hypothetical-technical as well as the hypothetical-pragmatic imperative. This is why I chose a new notion and call the pragmatic employment of reason the instrumentalist employment of reason or instrumentalist reasoning. Using the notion of an instrumentalist reasoning, furthermore, draws attention to the following aspect: instrumentalist reasoning asks for right means to given ends or to ends that are not fundamentally challenged. It does not ask for right ends and not for what is morally right. Given this definition, I show that an instrumentalist reasoning underlies a typical kind of interpretation of Habermas’s argument from human nature—the so-called instrumentalist reading. It takes Habermas to want to preserve human nature—an unchallenged end—and interprets him to evaluate germline editing not only as an inadequate means to preserve this end but to be a threat to it. It then attempts to show that Habermas’s argument works with a wrong—essentialist and determinist—notion of human nature, which subsequently leads to an inadequate evaluation of germline editing as means to change it.

Habermas’s reference to an ethical employment of reason does not find an analogy in Kant’s distinction of the three imperatives. The author of reference here is Aristotle and an ethics of the good life (2001, 4). The ethical employment of reason inspires an ethical reading of Habermas’s argument from nature, which I introduce as an alternative to the instrumentalist reading below.

## Genetic Essentialist and Determinist Objections to Habermas’s Argument From Human Nature —Plausible Within an Instrumentalist Reading?

There have been many critical remarks concerning Habermas’s appeal to nature. For the most part, they concern the relationship between genetic contingency of human procreation and perceived autonomy and equality. Here, I will focus on the attempt of critics to show that Habermas’s argument from nature wrongly asserts essentialist and genetic determinist premises. According to Elizabeth Fenton, Habermas wrongly assumes that human nature is something fixed and stable and that the normative claim to preserve it must fail. As technological interventions to what is considered a fixed human nature challenge exactly that idea, “it seems that we cannot be certain that human life as it is currently lived simply is the way it ought to be” (2006, 39). Fenton’s critique is therefore twofold. First, she rejects the idea of a status quo of human nature due to possible biotechnical interventions. Second, she discerns a case of naturalistic fallacy in Habermas’s argument. Both of Fenton’s objections do not apply to Habermas’s work. For one thing, he sees clearly the possibility of changing human nature in the sense that the contingently given genetic setup of humans might be changed by genetic engineering. For another thing, he does not simply demand to preserve human nature in order to keep a status quo. He even does not conceive of human nature as something “intrinsically valuable” as Fenton claims (2006, 41). Habermas clearly states that he wants to refrain from some “dubious sanctification” or “reenchantment of inner nature” (*FHN*, 5, 25). His specific form of “moralizing nature” is rather meant as an “assertion of an ethical self-understanding of the species which is crucial for our capacity to see ourselves as the authors of our own life histories, and to recognize one another as autonomous persons” (*FHN*, 25). The normative aspect of the notion of human nature is thus derivative insofar as genetic contingency and uncontrollability serve as a requirement for an autonomous and egalitarian human self-understanding. Because the latter is considered a condition for moral practices at all, that kind of normativity transfers back to its condition, namely human nature (*FHN*, 28).

Recently, several authors have resumed the objection of genetic determinism and essentialism with reference to empirical sciences. Oliver Feeney calls arguments from human nature that speak against enhancement as grounded in a myth “without sufficient empirical basis,” which leads to “groundless speculation and overreactionary normative responses” (2019, 237, 234). Because they are supposed to rely on genetic essentialist or determinist assumptions, those arguments are seen as underestimating the interplay of genetic and cultural social influences on human development (2019, 239). According to Sruthi Rothenfluch, Habermas’s argument is not in alignment with current findings on genetic function. She cites genetic studies implicating that neither genetic nor social influence alone determines a child’s identity. Due to the complex and variable interaction of both, there always remains an element of newness within a child’s development (2017, 2866f.). Timothy Murphy endorses the same thought when holding that “our contingency is unfinished and unfinishable” (2014, 338). Similarly, Nicolae Morar argues that Habermas’s notion of human nature wrongly presupposes kind essentialism and that his rejection of genetic engineering for purposes of enhancement rests on the problematic claim that “genetic modifications predetermine the future of enhanced children” (2015, 102f.). This misrepresentation of the relationship of genetic human nature and personal identity is thought to be due to reluctance to scientific findings in evolutionary biology and empirical psychology on Habermas`s side. Morar, for his part, refers to biological theory to prove wrong the idea that there is a common genetic setup that all and only humans share. Furthermore, he refers to psychological studies that do not support the assumption that genetically modified human beings are affected in their social, psychological, and emotional development (2015, 110).

While Habermas clearly rejects a concept of human nature as intrinsically valuable, his remarks concerning the relationship between genetic contingency and an autonomous and egalitarian human self-understanding unfortunately remain ambiguous. On the one hand, he holds that natural “contingency proves to be—in the very moment we can master it—a *necessary presupposition* for being-able-to-be-oneself and for the fundamentally egalitarian nature of our interpersonal relationships” (*FHN*, 13, my emphasis). A genetic determinist interpretation therefore does have some textual evidence; nevertheless, in the same book, Habermas also makes claims that are more moderate when he evaluates the impact genetic interventions could have on personality formation. He states that “the egocentric intervention takes on the meaning of a communicative action which *might* have existential consequences for the adolescent” (Habermas’s emphasis) and that “knowledge of the temporal prius of being made does not necessarily result in self-alienation” (*FHN*, 51, 54). Therefore, there is a more charitable interpretation, which does not support strict genetic determinism. Notwithstanding, even within this charitable reading, Habermas still brings forward reservations against genetic engineering for purposes of enhancement: “As long as we cannot be sure that this harmony between one’s own intentions and those of a third party will inevitably be produced, we cannot rule out the possibility of dissonant cases” (*FHN*, 61).

What becomes obvious at this point is that the above disagreement about the normative force of Habermas’s appeal to nature is about the burden and scope of proof. On the one hand, critics claim that Habermas’s reasoning relies on problematic premises. Genetic determinism has been widely rejected, and psychological studies do not seem to confirm a close connection of genetic contingency and perceived autonomy as of yet. According to the more charitable reading, on the other hand, ruling out strict genetic determinism is not sufficient to turn down Habermas’s concerns about genetic engineering for purposes of enhancement. The pure possibility that there might be cases in which genetically engineered individuals will not confirm their parents’ genetic intervention is reason enough for him to hold on to the claim that human nature is normative in the sense of it being a possible condition of the perception of autonomy and equality. As long as the pure possibility of dissonant cases is not ruled out, one should refrain from using the technology for purposes of enhancement.

In order to resolve the dispute between the two parties, one would have to ask: do we need proof that there is a possible connection between genetic contingency and perceived autonomy and equality in order to protect human nature? Or do we rather need proof that such a relation does not exist to rebut this argument from nature? Either way you look at it, the normative analysis at this point aims at an evaluation of the empirical consequences of using the technology, without discussing the ends for its use. I therefore argue that both—the critical reading underlying essentialist and determinist objections as well as the charitable reading, which sees Habermas as making a more moderate claim—operate within an instrumentalist employment of reason. This kind of reasoning is concerned with finding adequate means to undebated goals by solving empirical questions as well as by deciding on adequate technology. The critical instrumentalist reading does exactly that: it refers to empirical sciences as well as to technological possibilities in order to rebut reservations against germline editing. The end itself, the protection of human nature from enhancement interventions, is not itself debated. Fenton (2006) and Feeney (2019), for example, do not understand their arguments against Habermas to be support for enhancement. All they want to show is that Habermas’s reservation against enhancement does not follow from his argument from nature.

The charitable reading of Habermas’s argument operates at this level as well: it is concerned with the empirical possibility of disagreement between future children and parents resulting from the usage of germline editing. According to this charitable reading, germline editing is a problematic means because consequences of its use are uncertain. The argument itself is not about the evaluation of the possible end itself. The end—change of human nature for enhancement purposes—is already supposed to be problematic within this interpretation.

Within the instrumentalist use of reason, the question “What should I do?” is a matter of deciding empirical questions and choosing appropriate means. In case of human germline editing, answers to these questions remain rather speculative now, considering the undue risks of clinical germline editing (Lander et al. 2019). I therefore conclude that any argument relying on this kind of instrumentalist reasoning must remain rather weak. This applies to the critical assessment of Habermas’s argument from nature as brought forth from Fenton, Feeney, Rothenfluch, Murphy, and Morar as well as to the charitable interpretation, according to which Habermas makes a more moderate claim about possible consequences of using human genetic engineering.

## An Alternative Interpretation of the Argument From Human Nature—the Ethical Reading

If the instrumentalist reading of Habermas’s argument remains rather weak, is there an alternative reading that does not rely on speculative assumptions about the relation between genetic interventions and the development of specific character traits? I suggest that a better reading of the argument from human nature results when interpreted within the framework of an ethical use of reason. Within the ethical use of reason, the question “What should I do?” aims at figuring out fundamental personal interests and values. It refers to existential questions of the good life and a self-understanding of a person who tries to establish her identity. There is textual evidence that Habermas situates his argument from human nature within this framework, both at the level of the individual as well as at the level of society. The question his argument addresses within this ethical reading is therefore not whether germline editing is a technology whose use has genetic determinist implications. The relevant issue is much more if individuals, as well as humankind as a species, want to shape processes of procreation such that parents gain freedom of choice over the genetic setup of their children, provoking an experience of loss of contingency, autonomy, and equality.

There are three reasons that speak in favour of the ethical reading of Habermas’s argument from nature. First, his distinction of therapy and enhancement, second, his remarks on a shift from chance to choice, and third his account of an ethics of the species.

### The Distinction of Therapy and Enhancement

While Habermas’s argument from human nature speaks in favour of a conservative attitude towards human genetic engineering in general, he does approve of a restricted use of this technology for purposes of therapy as opposed to purposes of enhancement. It is therefore not genetic engineering per se, as a means, which is critically assessed by Habermas but the different purposes for which it can be used: “The problem, of course, is not genetic engineering, but the mode and scope of its use. It is, moreover, the *attitude* in which interventions in the genetic makeup of potential members of our moral community are carried out that provides the standards for an assessment of their moral admissibility” (*FHN*, 43, Habermas’s emphasis). What would be an attitude that allows for a morally admissible use of germline editing? According to Habermas, it is an attitude that is dedicated to the logic of healing. The logic of healing, including preventing severe diseases, allows for morally admissible uses of technology because establishing health is considered to be a common good by Habermas. As a common good, it is mediated by communicative processes within a moral community. The challenging task of a moral community is therefore to develop convincing criteria to distinguish healthy from sick forms of bodily existence in order to identify legitimate uses of genetic engineering in humans (*FHN*, 43f., 52).

Because there is supposed to be a common logic of healing, Habermas argues that one can assume consensus of a future person in case of therapeutic interventions; however, for purposes of enhancement, “virtual consent” of the child cannot be assumed, because it is impossible to foresee the child’s preferences. What is more, in this case, an intervention “according to the sole preferences of a third person” would be made, taking “on the form of an instrumentalization of human nature” (*FHN*, 52).

Habermas’s distinction of therapy and enhancement and the moral demands he deduces from it allow for a critical discussion themselves. One might object, for example, that it is difficult to rule out cases of dissonance not only in case of enhancement but also in case of therapy. For the purpose of my argument, however, what is important to see at this point is that this distinction speaks in favour of an ethical reading of his argument from nature. What becomes obvious is that Habermas does not make claims about the moral admissibility of the technology as such but about different purposes for its use; therefore, the argument refers to fundamental interests and values of individuals and a group of people and touches on questions of the good life. The focus lies not so much on germline editing as a means to undebated goals but much more on goals themselves and whether they should or should not be reached by using this technology.

### The Shift From Chance to Choice

There is a second reason that speaks in favour of an ethical reading of the argument from human nature. Going back to Habermas’s remarks about possible negative impacts of genetic engineering on the self-understanding of individuals will help clarify it. As shown above, some textual evidence might suggest an instrumentalist reading of the argument, which is concerned with the empirical consequences of the use of germline editing; nevertheless, Habermas also makes clear that his critical assessment of the technology does not *primarily* refer to possible genetic determinations of specific character traits. What is more important to him is that the experience of contingency might be lost to an *awareness* of being created once a child can be born with a genetic setup chosen by its parents:

Irrespective of how far genetic programming could actually go in fixing properties, dispositions, and skills, as well as in determining the behavior of the future person, post factum knowledge of this circumstance may intervene in the self-relation of the person, the relation to her bodily or mental existence. The change would take place in the mind. (*FHN*, 53)

According to Habermas, a shift of awareness could take place irrespective of the actual causal influence a specific genetic setup might have on character formation. The mere fact that parents made an active choice is what could cause a problematic self-image of a child. Leon-Philip Schäfer has convincingly argued that even children who themselves have not been genetically engineered can experience this shift of awareness once the technology is available (2019, 1061). He provides textual evidence that Habermas argues that supplying the technology poses a general threat to all future individuals, independent of an actual intervention. This “argument of mere controllability” draws on Habermas’s remarks on a possible omission to apply the technology, once it has become available for liberal use:

Exercising the power to dispose over the genetic predispositions of a future person means that from that point on, each person, whether she has been genetically programmed or not, can regard her own genome as the consequence of a criticizable action *or omission*. (*FHN*, 82f., Habermas’s emphasis)

In comparison to genetically edited individuals, a non-edited child might feel disadvantaged and impaired in its sense of equality. By choosing to refrain from genetic engineering, parents might therefore be confronted with the accusation that they did not use all the available means to augment certain features in the child.

The challenge of mere controllability has a follow-up-challenge. Robert Sparrow (2019) has recently pointed out that if enhancement procedures via germline editing steadily improve, then earlier enhancements will be obsolete compared to newer ones. This is why the project of parents to enhance specific traits of their children according to their conception of the good would, in some sense, always fail. Their child would be inferior to children born later with yet better enhancements. While Sparrow focuses on the ontological implications of obsolescence, one can easily see how psychological and societal challenges might arise. The awareness of having an obsolete genetic genotype, which might result in an obsolete phenotype, is likely to be a challenge to any future individual.

Several open peer commentaries critically discuss Sparrow’s article. Amongst them are objections to Sparrow that accuse him of genetic reductionism and determinism from within an instrumentalist reading, for instance Savulescu (2019b) and Chapman (2019).

Introducing the aspect of choice into the genetic lottery of procreation raises multiple possibilities to affect the self-understanding and identity of individuals. Habermas’s remarks on shifting the experience of genetic contingency to genetic choice therefore speaks in favour of an ethical reading of his argument from human nature.

### An Ethics of the Species

There is a third reason to interpret Habermas’s approach to genetic engineering from the perspective of an ethical employment of reason, and it is the most obvious one: he explicitly states that genetic engineering touches the ethical self-understanding of humans in its entirety, which is why an ethics of the species is needed. According to him, it is the overall structure of human moral experience that is at stake insofar as presuppositions of moral judgment and action in general might be affected by genetic engineering. These presuppositions include a self-understanding of moral individuals as autonomous and equal as well as being guided by norms and reason (*FHN*, 11, 28, 37ff.). Along with his claim that an ethics of the species is needed, Habermas therefore constrains a credo he otherwise endorses, namely the “priority of the just over the good” (*FHN*, 40). Because, for Habermas, germline editing touches the presuppositions of morality, coming to terms with an ethical evaluation of this technology is prior to questions of justice.

By claiming that genetic engineering touches general aspects of human self-understanding, Habermas transfers the ethical employment of practical reason from the individual level to the level of the species. At the level of the species, the ethical use of practical reason is not concerned with choosing between different values constitutive of one’s personal identity. At this level, the ethical use is about reflecting on anthropological characteristics that, for Habermas, comprise a self-understanding of individuals as autonomous and equal and that are shared and valued by all human beings (*FHN*, 39f.). This common image of what it is and should be like to be human, though intuitively clear to many, might not be as self-evident as Habermas claims. Daniel C. Henrich, while he opposes genetic determinist objections against Habermas, critically remarks that he lacks an elaborate normative anthropology or account of a good life. This is why his attempt to preserve our ethical self-understanding and with it human nature becomes rather decisionistic (2011, 263). For Vilhjálmur Árnason, however, Habermas already offers a more detailed image of what the human species is and should be. According to him, what lies behind Habermas’s rather speculative arguments about a possible determination of future persons is rather a defence of the communicative sphere of reason, which shall not be replaced by the demands of a profit-oriented market. Following Habermas, he warns about a “category mistake,” which consists in “employing technical solutions”—for example genetic engineering for purposes of moral enhancement—“to isolated parts of problems that need to be dealt with by political and pedagogical means” (2014, 365; see Bennett (2021) for a similar line of thought).

A thorough analysis of Habermas’s normative anthropology is beyond the scope of this paper. What is important to stress at this point is that Habermas’s remarks about an ethics of the species speak in favour of an ethical reading of his argument from nature.

## The Role of Social Aspects in Character Formation and Individual and Collective Self-Understanding

Along with objections to genetic essentialism and determinism, a critical instrumentalist reading often also raises another objection: the underestimation of social impacts on the development of a personal identity. Morar, for instance, argues that Habermas’s reservations against genetic enhancement makes little sense, considering the importance of processes of socialization (2015, 103). Feeney sees an “urgent need” to integrate more thoroughly the “role for sociological input (i.e. the pervasive effects of social structures) into the normative debates of genome editing” (2019, 242). Peter Herissone-Kelly rejects Habermas’s genetic determinism with reference to “the influence of others on our life histories” (2012, 202).

Objections of this kind are remarkable, considering Habermas’s elaborate work on the theory of communicative action and discourse ethics (1984/1987; 1990). But even if one remains within his reasoning in *FHN*, it becomes obvious that Habermas refers to the great impact social relations have on the building of a personality:

Since man, biologically speaking, is born “unfinished” and subject to lifelong dependency on the help, care, and respect of his social environment, individuation by DNA sequences is revealed as incomplete as soon as the process as social individuation sets in. Individuation, as part of life history, is an outcome of socialization. […] It takes entrance in the public sphere of a linguistic community for a natural creature to develop into both an individual and a person endowed with reason. (*FHN*, 34f.)^5^

The importance Habermas places on “relations of mutual recognition of communicatively acting persons” (*FHN*, 35) for individual and societal identity formation are also evident in the above introduced three reasons that speak in favour of an ethical reading of his argument from nature. The distinction of therapy and enhancement, the shift from chance to choice, and an ethics of the species connect ethical evaluation of germline editing to social structures as follows.

Habermas’s different assessment of genetic engineering for purposes of therapy and enhancement relies on social and communicative processes in at least two ways: for one thing, he considers it the task of a moral community to establish criteria to distinguish between the two purposes of intervening into the human genome. For another thing, he considers the notion of health, which underlies the distinction of therapy and enhancement, to be a common good. As a common good, it is subject to public discourse. So while Habermas rejects interventions for enhancement purposes due to concerns of merely individually cultivated preferences of parents, he accepts therapy interventions because they relate preferences of parents to preferences that are justified within a broader moral community. To say that Habermas underestimates social aspects in his approach to genetic engineering is therefore not very well grounded.

Recalling the other paring of words reinforces this conclusion. Habermas’s remarks about a possible shift from chance to choice and about possible changes in the self-understanding of individuals also refer to structures at the level of society. For one thing, he understands the impact parents have by choosing genetic interventions for their children primarily in psychological and sociological, not so much in biological terms: going back to a quotation from above: “The egocentric intervention takes on the meaning of a *communicative action* which might have existential consequences for the adolescent” (*FHN*, 51, my emphasis). For another thing, the argument from mere controllability shows that children that are not genetically modified might experience the non-intervention as an omission *in comparison to other children* who have been modified. Habermas’s normative claims therefore do not in the first place address relations that might or might not hold between certain genetic conditions and specific character traits. His evaluation of the technology draws much more on developments that occur at the level of society.

Social structures are furthermore essential to establishing an ethical self-understanding of the species. According to Habermas, it is especially the communicative sphere of reason, established by language-using agents, that constitutes this kind of species ethics. So both, germline editing as a form of communicative action as well as a species ethics are within the “logos of language,” which “embodies the power of the intersubjective” (*FHN*, 11). It is therefore evident that Habermas’s argument from nature does not neglect processes and structures of socialization, as some critics claim. Much to the contrary, it refers to social structures, when a) considering germline editing as a biogenetic technique that can be used for different purposes, b) when evaluating impacts that using this technique has on individuals, and c) on the human species.

As I have shown, an ethical reading puts objections to Habermas’s argument that arise within an instrumentalist reading into perspective: it is not plausible to interpret the argument as relying on genetic essentialist and determinist premises. It is furthermore wrong to accuse his approach of neglecting social aspects when evaluating genetic engineering. Much to the contrary, an ethical reading highlights how the evaluation of germline editing touches on fundamental value decisions as well as on the ethical self-understanding of individuals and humankind. Reference to the social structures is central for Habermas in this regard.

I would like to close by pointing to a conceptual challenge that arises within the ethical reading of Habermas’s argument. It relates to his distinction of genetic engineering and education. Habermas separates the two due to the irreversibility of genetic engineering. Given that an ethical reading of Habermas’s argument does not build on genetic determinist assumptions about character formation but refers much more to the level of social interaction, how does this distinction of education and genetic engineering still make sense? I see two options. The first is that the ethical reading must include empirical assumptions about the impact genetic interventions have on identity formation and self-understanding, even if genetic determinism is rejected. In this case, Habermas’s argument is in need of a more detailed empirical account of how exactly certain biological conditions can irreversibly influence the self-understanding of an individual. The drawback of this option is that it must rely on empirical speculation about the impact genes have on character formation. The second option I see is to refrain from reference to speculative biological assumptions and deny that the distinction of education and genetic intervention supports his argument in the sense described above. In this case, both education and genetic engineering are understood as forms of parental influence that reveal specific, negotiable conceptions of the good life. Since, for Habermas, the distinction between therapy and enhancement draws the line between legitimate and illegitimate uses of the biotechnology and since ruling out dissonance is the main criterion to distinguish the two, Habermas cannot offer a coercive argument against enhancement. For, if a moral community decides to include genetic enhancement as part of a good life, which is expected to be agreed upon by future individuals, then the use of germline engineering for purposes of enhancement would be ethically acceptable. Habermas himself admits that he is not offering a “conclusive moral argument” but rather “an orientation relying on an ethics of the species, which urges us to proceed with caution and moderation” (*FHN*, 29). While the ethical reading reminds us that germline editing touches on fundamental human values that lie beyond a means-ends rationality, it remains an open question whether germline editing as a communicative act indeed subverts presuppositions of moral judgment and action. Asking and engaging in answering it at the level of society is well worth it.^6^

## Conclusion

In this article, I show how genetic essentialist and determinist objections to Habermas’s argument from human nature originate from an instrumentalist reading, which, though there is some textual evidence for it, is neither very plausible nor very convincing. I draw on Habermas’s distinction of a threefold use of practical reason and provide three reasons from within his approach that speak in favour of an ethical reading of his argument. This reading not only avoids essentialist and determinist objections but also fundamentally incorporates social aspects of character formation and of individual and collective self-understanding. I therefore argue that it offers exactly what the critics claim is missing in Habermas’s account.

## Footnotes

1 The term “human germline editing” very broadly refers to introducing heritable changes into human DNA. Emmanuelle Charpentier and Jennifer Doudna introduced the CRISPR-Cas9 system in 2012. As early as 2018, He Jiankui announced the controversially discussed birth of twin girls whose germ line was edited using this technique.

2 See for example the change in evaluation of germline editing by the German Ethics Council. In its “Ad hoc recommendation” (2017), it very generally refers to germline editing as a morally “highly controversial technology” (3). Two years later, the Council published an “Opinion” (2019), which holds that germline editing is currently ethically untenable due to unforeseeable risks; nevertheless, it concludes that there is “no categorical inviolability of the human germline” (36) and asks for an international moratorium on the clinical application of the technique in order to discuss legitimate goals for possible interventions, given its safety and efficiency.

3 I will not discuss general concerns with arguments from nature that relate to the ambiguity and arbitrariness of the notion of “human nature” or to the naturalistic fallacy. I believe Habermas’s notion of human nature can be specified sufficiently and it will become obvious below that it is not prone to a naturalistic fallacy. For a general critique of arguments from nature, see, e.g., Kurt Bayertz (2003). For a negative critique in the context of genetic engineering/human enhancement, see, e.g., Nick Bostrom (2005); Allen Buchanan (2011, chapt. 4); Julian Savulescu (2019a). For a positive account of arguments from nature in the context of genetic engineering and enhancement, see, e.g., Hans Jonas (1984); Francis Fukuyama (2002); Leon Kass (2003); Michael Sandel (2007).

4 Habermas’s denotation of an “ethical” and a “moral” employment of practical reason also differs from another well-established differentiation between morality and ethics, wherein ethics stands for the study of morality.

5 According to Dieter C. Henrich (2011, 263), two anthropological accounts are present in Habermas’s work, both of which focus on the role of social and cultural integration of individuals in their upbringing: George Herbert Mead’s concept of linguistically mediated individuation through socialization and the theory of humans as deficient beings (Mängelwesen, as Arnold Gehlen puts it).

6 Recently, Francoise Baylis (2019) has argued that broad societal participation in deciding how to proceed with germline editing is essential. How this is not yet the case is shown by Gregor Wolbring and Lucy Diep (2016), who depict how the perspective of disabled people is mis- and underrepresented in discussions about genetic interventions in humans.
